# MLEE: A method for extracting object-level medical knowledge graph entities from Chinese clinical records

**DOI:** 10.3389/fgene.2022.900242

**Published:** 2022-07-22

**Authors:** Genghong Zhao, Wenjian Gu, Wei Cai, Zhiying Zhao, Xia Zhang, Jiren Liu

**Affiliations:** ^1^ School of Computer Science and Engineering Northeastern University, Shenyang, China; ^2^ Neusoft Research of Intelligent Healthcare Technology, Shenyang, China; ^3^ School of Computer Science and Technology, Harbin Institute of Technology, Harbin, China; ^4^ Department of Clinical Epidemiology, Shengjing Hospital of China Medical University, Shenyang, China; ^5^ Neusoft Corporation, Shenyang, China

**Keywords:** knowledge graph (KG), medical entity extraction, natural language processing (computer science), EMR data mining, Chinese clinical records

## Abstract

As a typical knowledge-intensive industry, the medical field uses knowledge graph technology to construct causal inference calculations, such as “symptom-disease”, “laboratory examination/imaging examination-disease”, and “disease-treatment method”. The continuous expansion of large electronic clinical records provides an opportunity to learn medical knowledge by machine learning. In this process, how to extract entities with a medical logic structure and how to make entity extraction more consistent with the logic of the text content in electronic clinical records are two issues that have become key in building a high-quality, medical knowledge graph. In this work, we describe a method for extracting medical entities using real Chinese clinical electronic clinical records. We define a computational architecture named MLEE to extract object-level entities with “object-attribute” dependencies. We conducted experiments based on randomly selected electronic clinical records of 1,000 patients from Shengjing Hospital of China Medical University to verify the effectiveness of the method.

## 1 Introduction

Since Google proposed the concept of a knowledge graph in 2012, it has become one of the hottest technologies in knowledge reasoning. An increasing number of researchers use the “entity-relationship” approach to express the real world (Zheng et al., 2021). This kind of knowledge representation has achieved perfect results in a search engine, question and answer (Q&A) format, etc. Various vertical fields are building more innovative application scenarios based on knowledge graphs. As a typical knowledge-intensive industry, healthcare is a popular vertical field that utilizes knowledge graph technology (Shi et al., 2017).

The shortage of global medical resources caused by Coronavirus Disease 2019 (COVID-19) has become a global disaster. Improving the medical efficiency of healthcare has become an urgent problem that needs to be solved by researchers worldwide (Zhu et al., 2017). Historically, many researchers have attempted to help doctors build a medical base and improve clinical efficiency (Jonnagaddala et al., 2015; Li et al., 2020b). Knowledge graph technology is currently a popular research direction in this field.

In medical knowledge graph technology, the first and most crucial step is to build a high-quality medical knowledge graph. In this step, researchers need to discuss the main issues from two perspectives: the data source for constructing the medical knowledge graph and the algorithm for extracting entities and relationships.

Data sources are divided into two types: data sources that use authoritative knowledge bases and data sources that use clinical record data. Building a knowledge graph based on a traditional knowledge base can usually ensure the accuracy of the data source because its knowledge is neatly organized. Although building a knowledge graph using such data is easy, due to the large individual differences among patients in the real world, the basis for judgment in clinical diagnosis is relatively complex. Enumeration in authoritative knowledge bases is challenging (Abhyuday et al., 2020). In addition, the lag in the update of such knowledge bases is problematic for inference calculations such as clinical decision support systems (CDSS). With the development of medical informatization in recent years, an increasing number of electronic medical records (EMRs), laboratory information systems (LISs), and PACKS have been established, providing a massive data foundation for the use of clinical data analysis, modeling, and information extraction. When using clinical records to build a knowledge graph, all patient data are entered and updated in real time, ensuring the validity and diversity of real-world data (Mykowiecka et al., 2009). However, the use of clinical records to build a knowledge graph has difficulties. When doctors write clinical records in natural language, the complexity of the patient’s condition is difficult for machines to understand (Louise et al., 2010).

In the process of using algorithms to construct a medical knowledge graph, in addition to using crawler technology to obtain data from a medical knowledge base with a relatively regular presentation structure (Li et al., 2020a), another technical route mainly uses deep learning to achieve both entity extraction and entity-relationship extraction. Relation extraction is a classification calculation in most research processes, and deep learning can usually achieve very high accuracy. However, challenges still exist when extracting and calculating medical entity recognition. First, when doctors write clinical records, they are not recorded for analysis by algorithms. The content of the records is usually complicated by the complexity of the patient’s condition, which is a challenge for both feature conversion and information extraction (Kang et al., 2017). Second, the medical information cannot precisely express medical entities through simple strings due to its particularity. For example, for the “fever” entity, multiple factors, such as the cause, occurrence time, duration, body temperature, and peak heat of the patient’s fever, need to be shown. When describing a patient’s fever, clinicians may even use only a description of the above information without using the word “fever".

The main contributions of this study are presented as follows:

By analyzing the relationship between clinical records and medical knowledge graphs, a set of methods for extracting medical entities from clinical data and constructing knowledge graphs is explored.

Through “punctuation correction”, the problem of entity recognition boundary errors caused by irregular medical records written by doctors is perfectly solved so that medical entities appearing in medical records can be stored in a complete semantic expression, avoiding information loss caused by the source.

Through clinical practice and data experiments, the hidden category attributes of sentences in medical records are verified, minimizing the semantic space of each category of medical entities during extraction, thereby improving the accuracy of entity recognition.

Last, two layers of basic sequence annotation calculation are used to extract medical entity fragments and entity attributes from the text to complete the extraction of medical entities from clinical medical records.

The clinical records are parsed by simulating how clinicians write records, and then medical entities are extracted.

The medical entity extracted by this method is a solid entity with “object attributes”. Such entities can be directly utilized to construct medical knowledge graphs and can serve as input data for knowledge graph reasoning calculations. By increasing the diversity of information within entities, reasoning accuracy using knowledge graphs is improved.

The remainder of this paper is organized as follows: The second chapter introduces the current methods from researchers to construct medical knowledge graphs and to extract medical entities from various types of data. The third chapter introduces the detailed process of extracting medical entities from clinical record data in this study. The fourth chapter introduces the experimental results of this method using actual clinical data to extract medical entities. The fifth chapter introduces the conclusions of this research and prospects for future work. The source code is available at https://github.com/cocojoe0220/MLEE.

## 2 Related work

Research on building knowledge graphs has become very popular in recent years—researchers complete entity recognition and entity-relationship recognition by constructing novel computational architectures (Uzuner et al., 2010; Weng et al., 2017; Zhao et al., 2017; Cheng et al., 2019; Qiu et al., 2019; Wu et al., 2021). Related research on medical data to build knowledge graphs is continually emerging. These studies focus on building knowledge graphs based on clinical medical record data and building knowledge graphs based on public medical health datasets (Jiang et al., 2017; Jiang et al., 2021).

Liu and Xu (2021) attempt to build a knowledge graph from real-world, “dirty” electronic medical records. In this study, after extracting “symptom-disease"-related data from clinical medical records, the medical record text itself is used to complete disambiguation based on similarity calculation and to construct a knowledge graph related to symptoms and diseases. The disease prediction calculation based on patient symptoms is completed based on the knowledge graph. Weng et al. (2017) (Weng et al., 2017)used traditional Chinese medicine (TCM) unstructured clinical text data, clinical protocol guidelines, medical textbooks, and other data to construct a TCM clinical knowledge graph based on the triad structure. This research describes an entity through the Resource Description Framework (RDF) and combines the relationship between TCM and human body parts to construct an entity with upper and lower relationships and forms a complex network of directed knowledge elements. This approach reflects the potential logical relationship between knowledge elements in TCM. Wu et al. (2021) used public medical quiz information and encyclopedia data on the Internet. The researchers proposed the co-training double word embedding conditioned bidirectional long short-term memory (CTD-BLSTM) computing architecture to improve the accuracy of medical named entities and entity relationships in the Chinese field and to provide better support for constructing a Chinese medical knowledge graph.

We have summarized and discussed the current related research on the construction of medical knowledge graphs and discovered that most researchers usually analyze the problem from the perspective of computer practitioners when conducting research. From the triad structure born from the knowledge graph until now, researchers in the industry have proposed the tuple data structure. These studies always use algorithms to achieve better computational accuracy and more diverse ways of reasoning. Just as doctors need to obtain multidimensional information in evidence-based medicine to diagnose diseases, medical entities also need multidimensional information to be fully expressed. We do not suggest that an ordinary triad can express the complete relationship between two medical entities. For example, the relationship between “fever-cough” and “fever-body temperature” or “fever-duration” are not in the same dimension. Building a knowledge graph from clinical data requires deeper data structures and computational architectures.

## 3 Materials and methods

The electronic clinical record covers the patient’s condition and the diagnosis and treatment process. A point worthy of discussion is whether different doctors follow fixed rules when recording clinical records. Although we have not identified relevant rules and regulations in the medical industry, we have noticed that in the process of multidisciplinary treatment (MDT), clinicians from different departments, hospitals, and even countries can analyze a condition based on the same clinical record data. However, different clinicians can read the same clinical records, which also indicates that clinicians follow the rules of a fixed pattern in the medical industry when recording clinical records. Although this invisible rule should follow the basic logic of clinical diagnosis and treatment, it also standardizes the information presentation structure of clinicians when writing clinical records. This rule is the logic by which we extract medical entities from clinical records through algorithms.

**FIGURE 1:**
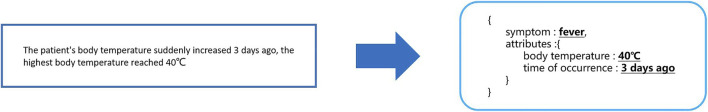
Extract fever-related attributes from the fever description segment.

By reviewing a numerous clinical records, we discovered that the logic of clinicians in writing clinical records is very clear. Consider the “Admission Record - Present Illness History”, which records the patient’s condition when they are admitted to the hospital as an example. Clinicians described the patient’s symptoms, treatment methods, key indicators of laboratory examinations, and imaging findings in several sentences in the clinical record text. Proceeding to the next level of analysis, in the description of the patient’s symptoms, the symptoms, degree, physical indicators associated with symptoms (such as recording body temperature during fever), cause of occurrence, time of occurrence, duration, aggravating factors, and mitigating factors. When describing the treatment method, for operation treatment, the type and date of the operation will be recorded; for medication treatment, the name of the drug, the dose, and the number of times will be recorded. A recording laboratory test will record the names and values of important indicators. The type of imaging examination, examination site, and abnormal findings will be recorded for imaging examination. These records can almost be the record rules that any hospital, department, and clinician will follow. The logical structure of these records is the same entity structure employed when we extract information. To extract medical entities from such clinical records, we can split them into the following process:

We want to extract medical entities that need to conform to medical logic and have an “object-attribute” structure. Therefore, we have to extract the entity’s attributes from the description related to each medical entity, as shown below in Figure 1.

**FIGURE 2:**
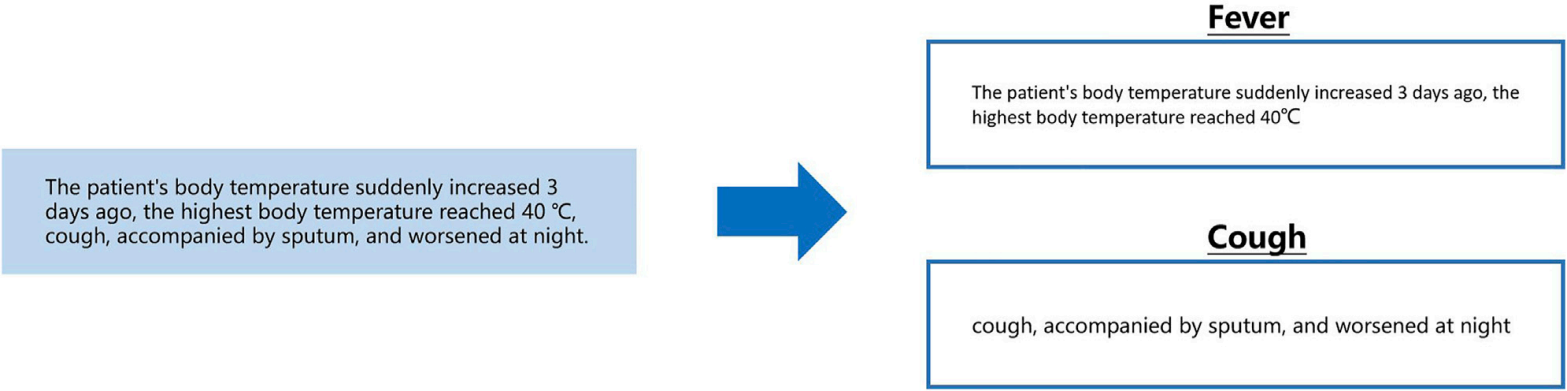
From a sentence describing symptoms, separately extract segments describing fever and cough.

In the above example, the text on the left is a segment from the clinical record text that describes the patient’s fever. Extracting “body temperature” and “occurrence time” from this segment can be performed by a sequence labeling algorithm. However, note that “body temperature” is unique to the symptom “fever”. When extracting this kind of information, it is necessary to know in advance that the current segment describes “fever”. When doctors describe patients’ symptoms, they usually make a centralized record in the same sentence. To obtain the fever description segment in the clinical records required for the above calculation, we designed a calculation as shown in the following Figure 2.

**FIGURE 3:**
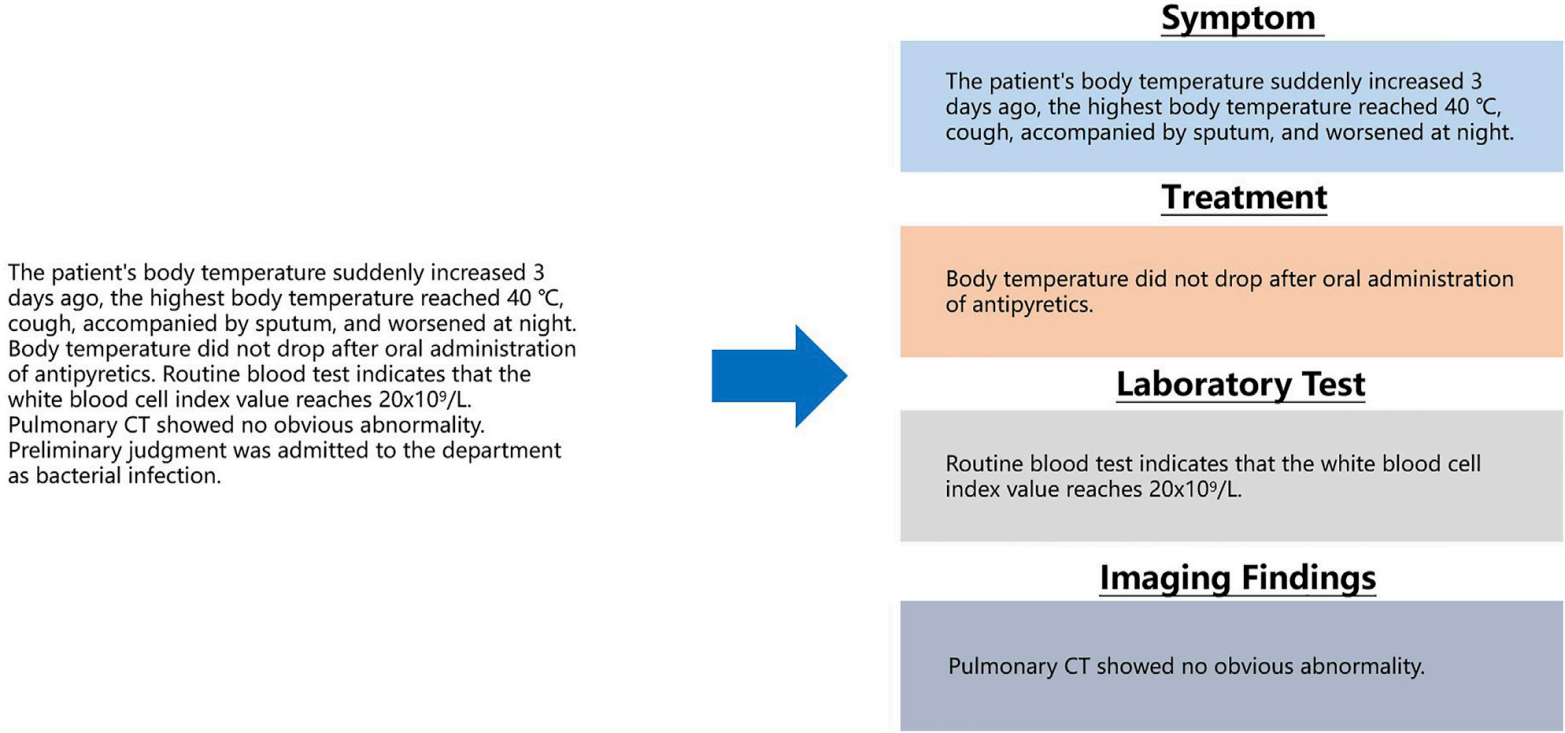
Classify sentences in clinical record text into correct categories.

The content shown in the above figure can be understood as the need to segment the description of fever and cough from a sentence describing a patient’s symptoms and to give corresponding symptom labels. This process can be conducted by long entity recognition in sequence labeling computation. The next problem then becomes that we need to classify the sentences in the text clinical records into a known category. As previously described, when recording the basic condition, clinicians usually use several fixed sentence patterns (symptoms, treatment methods, key indicators of laboratory examinations, and imaging findings). Using text classification computing to complete this task is a good choice as shown in the following Figure 3.

**FIGURE 4:**
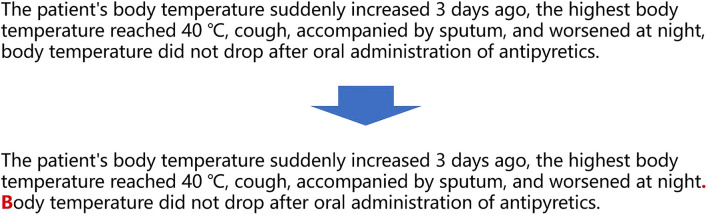
Correct misuse of punctuation in clinical record text.

As shown in the above figure, as long as the sentences in the text clinical records are calculated through the classification calculation, the corresponding categories of the sentences are obtained, and entity recognition and entity attribute recognition can be performed. However, in actual work, we discovered an easily overlooked detail. When Chinese clinicians write clinical records, punctuation is irregular, and even the entire clinical records are separated by commas. For this kind of irregularity, there is no hospital or relevant department to supervise. Although this irregularity does not affect human reading, for computers, this irregularity will produce low-precision classification calculations due to unclear sentence boundaries. To solve this problem, a punctuation correction calculation needs to be prepended before the clinical record sentence classification calculation as shown in the following Figure 4.

The above content describes the researcher’s final plan to use four steps to extract medical entities after analyzing the logic in the text clinical records. The four steps are arranged in positive order based on data processing, namely, “punctuation correction”, “sentence classification”, “medical entity extraction”, and “entity object attribute extraction".

### 3.1 Punctuation Correction

We obtained a random sample of 500 medical records from the EMRs of hospital departments. The count revealed that a total of 16,764 punctuation marks were utilized in these cases. According to the rules, we manually confirm the existing punctuation in the clinical medical record and correct the incorrect punctuation in the medical record. If manual correction was employed as the standard, the punctuation correctness rate for clinicians writing medical records was only 16.4%.

Based on this manually modified database, we plan to build a sequence annotation model. An elementary and effective neural network was constructed to accommodate the punctuation correction and subsequent information extraction. In the embedding layer, we chose to use the Bidirectional Encoder Representations from Transformers (BERT) model (Devlin et al., 2018). Although we initially tried to use Word2Vec for embedding based on a large amount of data, the results were approximately 4% lower than those based on BERT.

In building the actual sequence annotation, we made some changes to the original BERT, which processed tokens by slicing most characters. For example, we discovered that slicing words could sometimes significantly impact the meaning of Chinese expressions (Névéol et al., 2018). We therefore reworked the token in BERT to slice and dice by any individual character.

**FIGURE 5:**
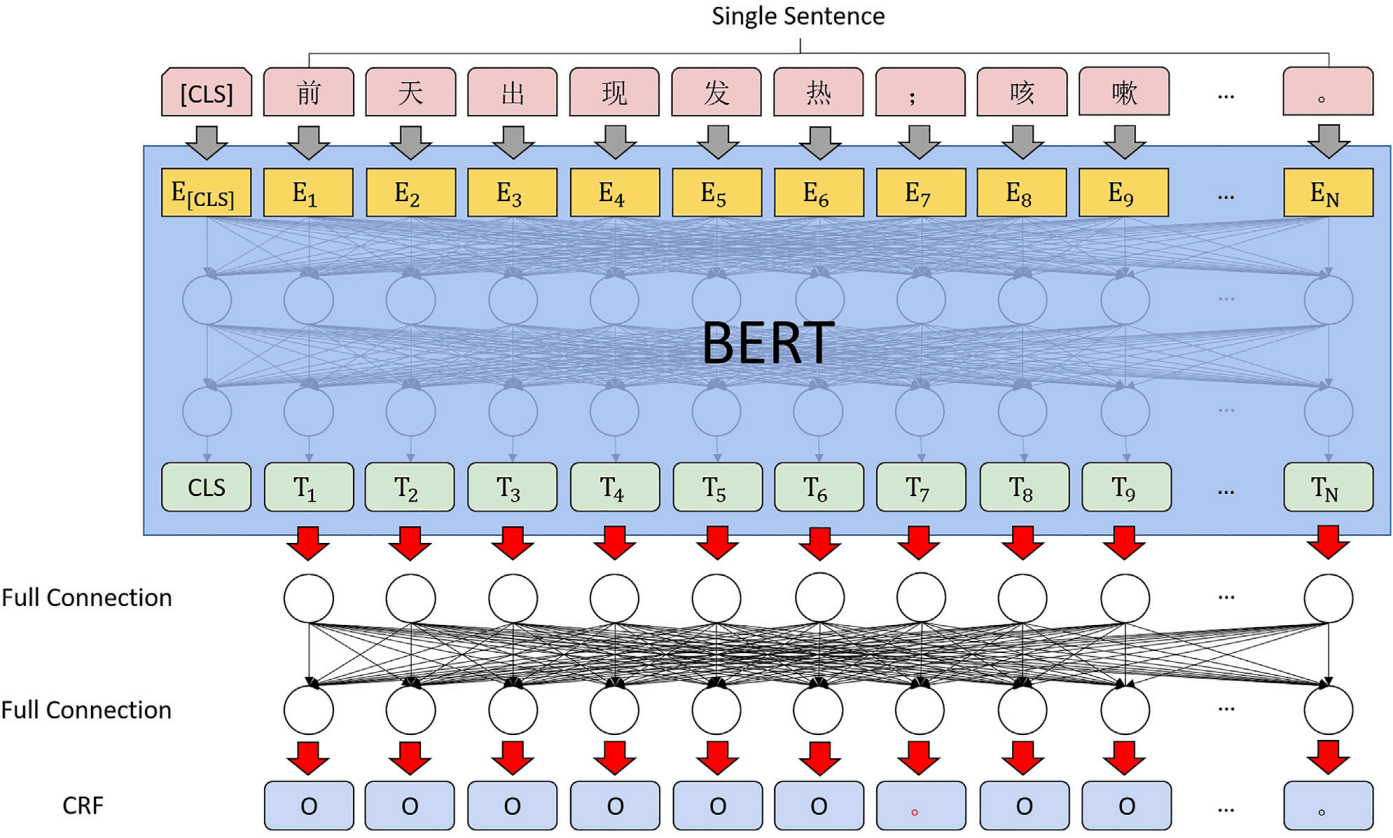
Punctuation correction computing architecture.

We tried to discard the long short-term memory (LSTM) (Greff et al., 2016) during the calculation of the sequence annotation of the correction markers. The transformer performs much better than the recurrent neural network (RNN) in many tasks. As Chinese words are stitched together from multiple characters, the profession usually uses the transformer’s output at the last encoder layer in BERT as input for subsequent docking of bidirectional LSTM with a conditional random field (Bi-LSTM + CRF) (Huang et al., 2015). However, since the sequence information in the transformer itself is sufficient, obtaining the sequence information of the context by using RNN (LSTM) again is unnecessary (Feng et al., 2018). We also wanted to give the neural network as much information as possible by appending a CRF after the last fully connected CRF. The computing architecture is shown in Figure 5.

### 3.2 Sentence Classification

According to the information obtained through the EMR system, the actual patient will generate 27 subcategories of clinical records.

After considering all types of clinical records, we discovered that the same types of sentences occur in many different types of medical record types. Treatment-related descriptions appear in the “past history”, “treatment plan”, “discharge instructions and rehabilitation instructions” and other types of medical records. If one follows this pattern, there must be a range of sentence types that can cover the semantic content of all types of medical records (Frunza and Inkpen, 2010). The clustering of all statements in the clinical records was calculated using the clustering calculation (Rodriguez and Laio, 2014), and the validity of the current clustering results was verified using the silhouette coefficient.

We then manually observed the clustering results, and after merging the two smaller clusters based on the semantics of the clinical history statements, we obtained 18 clusters. Afterward, the content of the utterances in each cluster was again manually and semantically confirmed, and medical semantic description labels were associated with each of these 18 clusters. This labelling includes a description of symptoms, treatment, signs and symptoms, specialist examination, examination information, etc.

**FIGURE 6:**
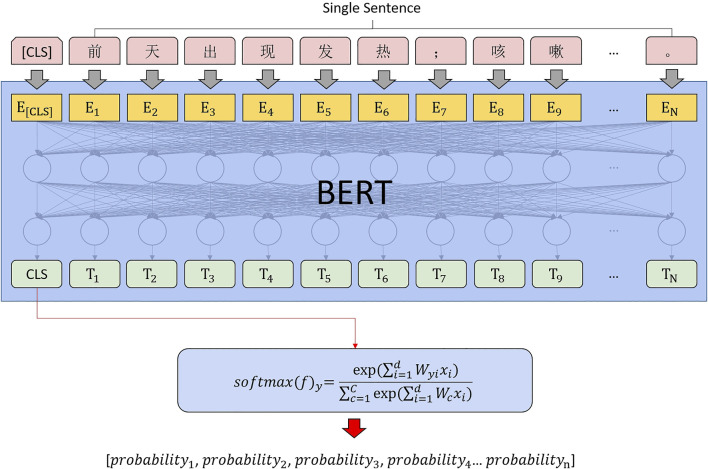
Sentence classification computing architecture.

We constructed a text classifier based on BERT + FC + Softmax (Kim, 2014) as shown in Figure 6; the model was validated in multiple rounds by cross-validation.

### 3.3 Medical Entity Extraction and entity Object Attribute Extraction

After completing punctuation correction and sentence classification, the final entity description segment extraction and entity attribute extraction process can be understood as a short text sequence annotation.

The semantic scope of entities and attributes in the medical field is relatively small, and the semantic space of the text to be extracted has been fixed through the above two steps, which is a very simple calculation scenario for sequence labeling.

**FIGURE 7:**
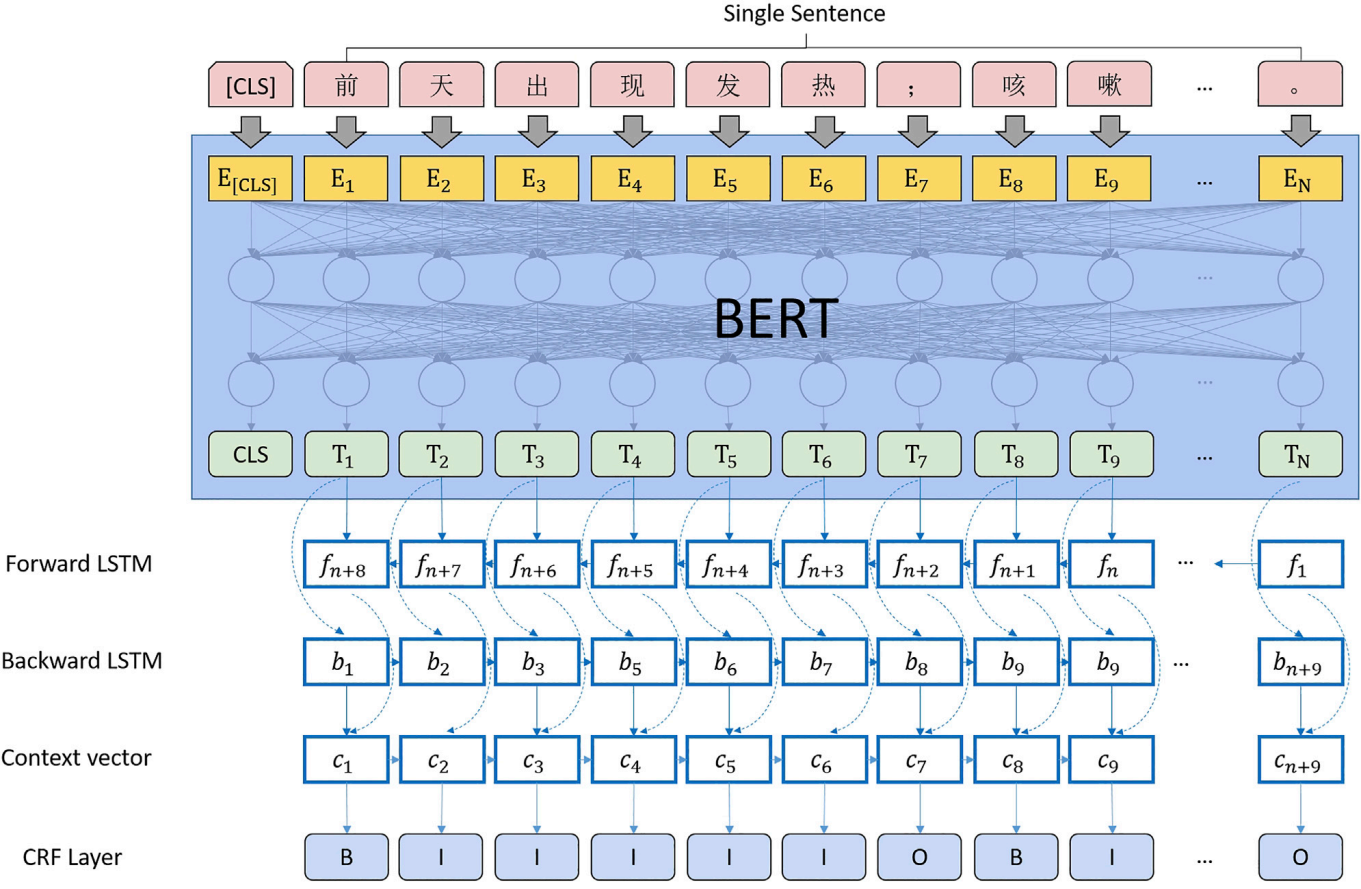
Entity extraction computing architecture.

Since the entire computing architecture needs to be merged to ensure the consistency of feature extraction, BERT + Bi-LSTM + CRF is selected for sequence annotation, as shown in Figure 7.

### 3.4 Computing Architecture

**FIGURE 8:**
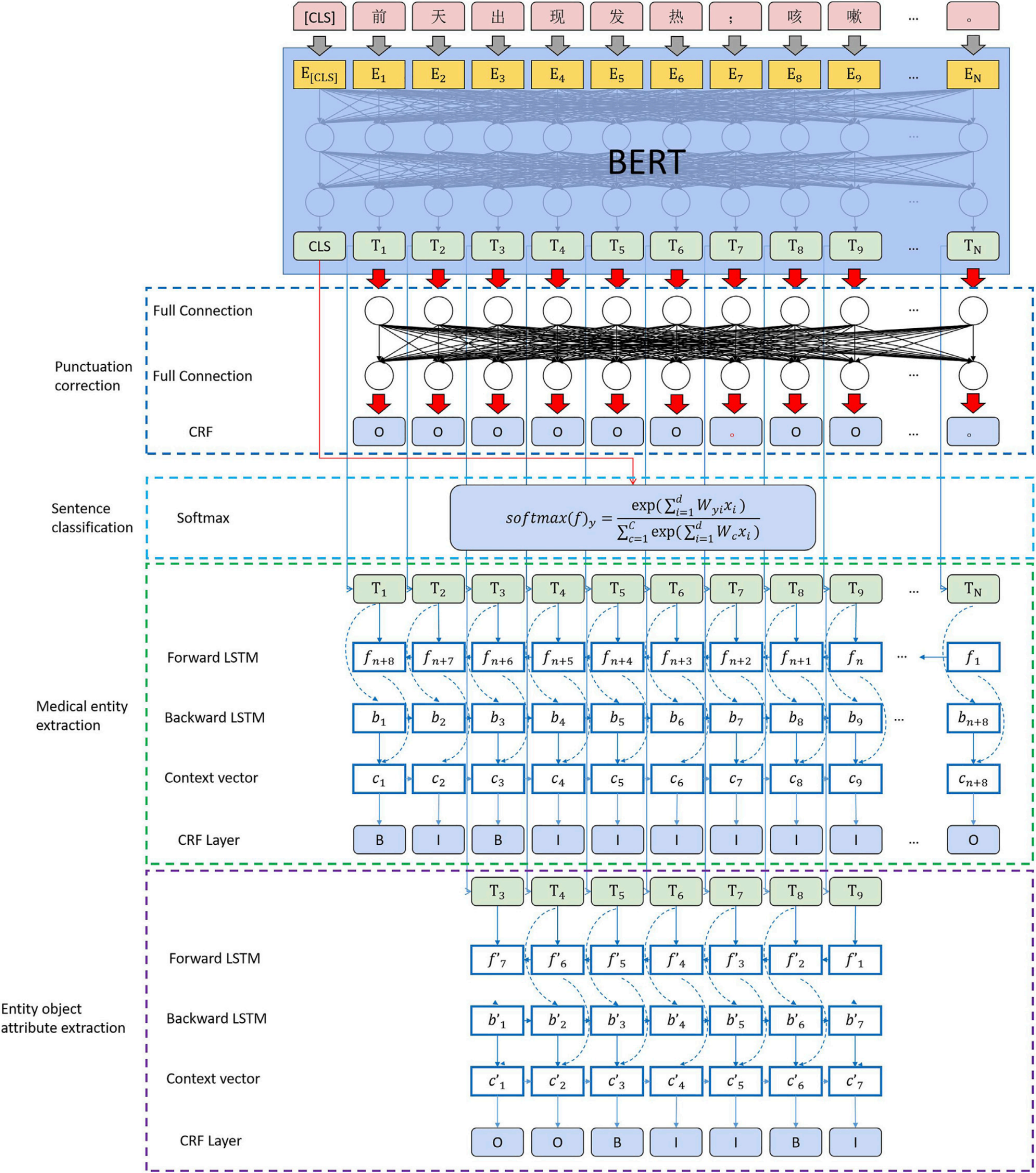
MLEE computing architecture.

We built the computing architecture, as shown in Figure 8. After using BERT to complete the feature conversion of text data, we realize the extraction and calculation of medical entities by connecting four downstream tasks. The detailed process is presented as follows:

1) Complete the punctuation correction calculation using a fully connected layer and conditional random fields.
2) Use the CLS vector generated by BERT for the sentence and complete the sentence classification through softmax.
3) Sequence annotation of medical entity segments using bidirectional LSTM and CRF.
4) Perform the final medical entity attribute extraction using bidirectional LSTM and CRF.

In this computing architecture, it is necessary to explain the change in the loss function of BERT in the upstream computing process in the multi-downstream task scenario.

$$\mathrm{Loss}\left(\text{θ},\tilde{{\text{θ}}_{1}},{\text{θ}}_{2}\right)=\mathrm{Loss}\left(\text{θ},\tilde{{\text{θ}}_{1}}\right)+\mathrm{Loss}\left(\text{θ},{\text{θ}}_{2}\right)$$ (1)

where θ represents the parameters of the Encoder part in BERT. $\tilde{{\text{θ}}_{1}}$ in the original BERT paper represents the parameters in the output layer connected to the encoder in the masked-language modeling (LM) task. This study represents the parameter combination of three sequence annotations after being output by the encoder. ${\text{θ}}_{2}$ The original paper represents the classifier parameters connected to the encoder in the sentence prediction task. This study represents the classifier parameters in the classification calculation of text medical record sentences. Details are presented as follows:

$$\text{Loss}\left(\text{θ},{\text{θ}}_{11}\right)=-\Sigma_{i=1}^{M}\log\;P\left(m=m_{i}|\text{θ},{\text{θ}}_{11}\right),m_{i}\in \left[1,2\ldots ,\left|\mathrm{Punctuation}\;\mathrm{Set}\right|\right]$$ (2)

where ${\text{θ}}_{11}$ represents the parameters in the output layer connected to the encoder in the punctuation correction sequence labeling task.

$$\text{Loss}\left(\text{θ},{\text{θ}}_{12}\right)=-\Sigma_{j=1}^{N}\log\;P\left(n=n_{j}|\text{θ},{\text{θ}}_{12}\right),n_{j}\in \left[1,2\ldots ,\left|\mathrm{Medical}\;\mathrm{Entity}\;\mathrm{Set}\right|\right]$$ (3)

${\text{θ}}_{12}$ represents the parameters in the output layer connected to the encoder in the medical entity description segment sequence labeling task.

$$\text{Loss}\left(\text{θ},{\text{θ}}_{13}\right)=-\Sigma_{k=1}^{N}\log\;P\left(o=o_{k}|\text{θ},{\text{θ}}_{13}\right),o_{k}\in \left[1,2\ldots ,\left|\mathrm{Entity}\;\mathrm{Attribute}\;\mathrm{Set}\right|\right]$$ (4)

${\text{θ}}_{13}$ represents the parameters in the output layer connected to the encoder in the medical entity attribute sequence labeling task.

$$\mathrm{Loss}\left(\text{θ},\tilde{{\text{θ}}_{1}}\right)=\mathrm{Loss}\left(\text{θ},{\text{θ}}_{11}\right)+\mathrm{Loss}\left(\text{θ},{\text{θ}}_{12}\right)+\mathrm{Loss}\left(\text{θ},{\text{θ}}_{13}\right)$$ (5)

The loss of the three downstream sequence labeling tasks is added to obtain $\mathrm{Loss}\left(\text{θ},\tilde{{\text{θ}}_{1}}\right)$ .

$$\text{Loss}\left(\text{θ},{\text{θ}}_{2}\right)=-\Sigma_{l=1}^{H}\log\;P\left(h=h_{l}|\text{θ},{\text{θ}}_{2}\right),h_{l}\in \left[\mathrm{labe}l_{1},\mathrm{labe}l_{2}\ldots ,\mathrm{labe}l_{x}\right]$$ (6)

In the second part, $\mathrm{Loss}\left(\text{θ},{\text{θ}}_{2}\right)$ is the loss function of the sentence classification task.

## 4 Experiment

This chapter introduces the experiment in three parts. The first part concerns data sources, the definition of medical entities in the schema, and data annotation. In the second, we introduce the extraction of medical entities based on the computational architecture proposed in this study. Since there is currently no open-source text clinical record dataset in the Chinese field and based on the diseases involved in the current clinical records (pediatric respiratory diseases), there is no unified knowledge map schema standard. This paper temporarily evaluates the effect based on the data extraction accuracy of the in-hospital data based on the data standard jointly constructed by the author and the clinicians of Shengjing Hospital of China Medical University. In the third part, we test all the entity attributes of the custom schema by flattening to test whether the computational architecture proposed in this study has an accuracy loss comparable with the general sequence annotation.

### 4.1 Data Preparation

We randomly selected the current illness histories of 1,000 patients from the inpatient clinical records at Shengjing Hospital of China Medical University. We discussed them with clinicians and learned about their concerns about writing and reading clinical records. Combined with the definition of medical fields in the Snomed CT International Edition, the medical entities and attribute labels in the schema are sorted, as shown Table 1.

**TABLE 1:** Medical knowledge graph schema label for information extraction.

Entity Type	Entity	Attributes
Symptom	Fever	Body Temperature
		Occurrence
		Duration
	Cough	Occurrence
		Duration
		Aggravating Factor
		Relieving Factor
		Cough Frequency
		Situation
Treatment	Medication Treatment	Drug name
		Drug dose
		Duration of course of treatment
	Operation	Type of operation
		Date of operation
		Adverse reactions
Laboratory Test	Laboratory Test Entity	Test item
		Value
Imaging	Computed Tomography	Body part
		Abnormal seen
	Magnetic Resonance Imaging	Body part
		Abnormal seen
		T1WI
		T2WI
Other		

Based on the above labels, we use “entity type” as the classification calculation label of medical record sentences, “entity” as the sequence annotation label of medical entity segments, and “attribute” as the sequence annotation label of medical entity attributes. In the process of punctuation correction, the “period” is corrected to ensure that these sentences can be correctly split. The data were labeled according to the table by clinicians and used as the gold standard.

According to the above rules, we manually marked 7,029 sentences (3,418 punctuation points were manually corrected, and the error rate of punctuation used by doctors reached 48.6%), 10,467 medical entities, and 29,478 medical attributes based on the clinical medical records of 1,000 patients. entities with 2.82 attributes).

### 4.2 Description of Effect

The above data and the entity labels defined in schema model training and effect verification are carried out based on the computing architecture introduced in the previous chapter. The calculation effect of all steps is presented as Table 2.

**TABLE 2:** Effect of each calculation step of MLEE.

Computational Procedure	Precision	Recall	F1 value
Punctuation correction	0.9874	0.9529	0.9698
Sentence classification	0.9812		
Medical entity extraction	0.9611	0.9438	0.9524
Entity object attribute extraction	0.9638	0.9611	0.9624

The experimental results exceeded our expectations, and we subsequently analyzed the calculation results by decomposing steps. Most of the miscalculated punctuation is concentrated in the over segmentation of symptom-related descriptions in the punctuation correction step. For example, “fever” and “cough”, which should be listed in the same sentence, are divided into two sentences. Such errors do not cause error propagation in subsequent computations. In the sentence classification step, because we built an “Other” category to carry some content in the clinical record about the patient’s general condition before admission, the patient’s body temperature, mental state, appetite, and other related information may be included. Some of these sentences are divided into “symptom” labels for the last two sequence annotation computations. Although the input of the final entity attribute sequence annotation labeling is the output of the previous layer of medical entity segment sequence annotation labeling, the error propagation will be critical. However, the results indicate that the accuracy of the lower layer calculation is higher than that of the upper layer calculation. The researchers determined that when calculating the medical entity segment, precision and recall may decrease due to the error of one character before or after. However, as long as it contains all the characters required for the lower-level sequence annotation labeling, the correct result can still be obtained in the final entity attribute calculation.

### 4.3 Calculate Loss Assessment

To evaluate whether the superimposed computing architecture of this study will lose accuracy through error transmission, we compare the accuracy by flattening the labels in the schema. The sequence annotation labels used for testing are shown in the last column of Table 3.

**TABLE 3:** Labels for flat transformation using the schema.

Entity type	Entity	Attributes	NER Label
Symptom	Fever	Body Temperature	**Fever-Body Temperature**
		Occurrence	**Fever-Occurrence**
		Duration	**Fever-Duration**
	Cough	Occurrence	**Cough-Occurrence**
		Duration	**Cough-Duration**
		Aggravating Factor	**Cough-Aggravating Factor**
		Relieving Factor	**Cough-Relieving Factor**
		Cough Frequency	**Cough-Cough Frequency**
		Situation	**Cough-Situation**
Treatment	Medication Treatment	Drug name	**Medication Treatment-Drug name**
		Drug dose	**Medication Treatment-Drug dose**
		Duration of course of treatment	**Medication Treatment-Duration of course of treatment**
	Operation	Type of operation	**Operation-Type of operation**
		Date of operation	**Operation-Date of operation**
		Adverse reactions	**Operation-Adverse reactions**
Laboratory Test	Laboratory Test Entity	Test item	**Laboratory Test Entity-Test item**
		Value	**Laboratory Test Entity-Value**
Imaging	Computed Tomography	Body part	**Computed Tomography-Body part**
		Abnormal seen	**Computed Tomography-Abnormal seen**
	Magnetic Resonance Imaging	Body part	**Magnetic Resonance Imaging-Body part**
		Abnormal seen	**Magnetic Resonance Imaging-Abnormal seen**
		T1WI	**Magnetic Resonance Imaging-T1WI**
		T2WI	**Magnetic Resonance Imaging-T2WI**

The bold values indicate NER label, it represents the label used to annotation the real data.

The final comparison accuracy is shown as Table 4.

**TABLE 4:** Comparison of MLEE information extraction and traditional sequence labeling.

Method	F1 value
Bert + BiLSTM + CRF	0.9367
**MLEE**	**0.9624**

The bold values indicate experiment results of the method proposed in this paper.

This conclusion also confirms that the method proposed in this study improves the information extraction accuracy compared with general sequence annotation and better expresses medical entities through the “object-attribute” structure. This finding provides a good data foundation for constructing medical knowledge graphs and reasoning computations based on knowledge graphs.

## 5 Conclusion

In this paper, we propose a method for extracting medical entities using real Chinese clinical medical records. A medical knowledge graph based on clinical data can be constructed on this basis. We discovered that the same medical record data, simply based on entity co-occurrence, can be used as a high-quality relational to connect entities. If many cases, the data can be utilized as the research object, even directed probability edges can be obtained, which is the follow-up research direction of the research team.

## Data Availability

The data analyzed in this study is subject to the following licenses/restrictions The data used in this study came from the hospital’s electronic medical record system. All data used in the experiment did not involve any personal information of patients, and all data experiments were carried out in the hospital. Requests to access these datasets should be directed to Zhiying Zhao, zhaozy@sj-hospital.org.
